# Tuberculosis, war, and refugees: Spotlight on the Syrian humanitarian crisis

**DOI:** 10.1371/journal.ppat.1007014

**Published:** 2018-06-07

**Authors:** Mohamad Bachar Ismail, Rayane Rafei, Fouad Dabboussi, Monzer Hamze

**Affiliations:** Health and Environment Microbiology Laboratory, Doctoral School of Sciences and Technology, Faculty of Public Health, Lebanese University, Tripoli, Lebanon; Tufts Univ School of Medicine, UNITED STATES

## Overview

Tuberculosis (TB) is a leading cause of mortality and morbidity worldwide. The spread of this fatal disease increases in crisis-affected populations. The ongoing Syrian civil war has led to significant damage to the national healthcare system and forced millions of Syrians to take refuge in neighboring countries, where the majority face miserable conditions. These circumstances increase the risk of TB development and spreading among Syrian refugees (SRs) and their host communities. After the beginning of the Syrian crisis in 2011, a remarkable increase in TB cases was reported in countries bordering Syria and is essentially attributed to the massive displacement of the SR population. Here, we first review the risk of TB dissemination during wars and among displaced populations, notably refugees. Then, we discuss the causes, current situation, and complications of the growing TB risk among SRs in Syria’s neighboring countries, focusing in particular on Lebanon. The aim is to highlight the spreading potential of this disease among vulnerable populations facing such complex conditions of conflicts and forced displacements.

## Armed conflicts and forced population displacements markedly increase TB risk and selection of TB-resistant forms

TB, caused by *Mycobacterium tuberculosis* (Mtb), is currently the first leading infectious killer. In 2016, the World Health Organization (WHO) estimated that 10.4 million people developed TB and about 1.7 million died from the disease. Classically, two TB-related forms exist: asymptomatic latent TB infection (LTBI) and symptomatic active TB disease. Unfortunately, global TB control is complicated by the emergence of drug-resistant TB such as MDR-TB (multidrug-resistant TB) and XDR-TB (extensive drug-resistant TB).

Crises, including armed conflicts and population displacements, are often associated with up to 20-fold increases in the risk of TB [1]. Indeed, wars and armed conflicts are powerful public health enemies that destroy basic medical infrastructure, hinder health agendas, hamper immunization programs, and cause significant shortages in healthcare workers and medicines [2]. Regrettably, the effects of war are not limited to areas where war occurs, since they also force huge numbers of people to be internally displaced or to take refuge in neighboring countries, where they generally experience unfavorable living circumstances that promote the spread of communicable diseases. Since World War I, the accumulation of evidence has clearly shown that areas where living conditions are disrupted by war represent an ideal environment for TB development and that refugees and conflict-affected and displaced populations serve as breeding grounds for the dissemination of this fatal disease and its resistant forms (Table 1).

**Table 1:** Impact of wars and population displacement on TB development, spreading, and resistance.

	Impact on TB development, spreading, and resistance	Reference
**War/war-torn country**		
World War I	A dramatic increase in TB mortality rates in war-involved countries (more than one million deaths in one year) majorly by conversion from LTBI to active TB	[3,4]
Bosnia-Herzegovina	A 4-fold increase of newly diagnosed TB cases	[5]
Guinea-Bissau	A 3-fold increase in TB mortality rates (12 versus 34 per 100 persons per year before and after the war, respectively)	[6]
Congo Brazzaville	A 2-fold increase in the number of TB patients	[7]
Afghanistan	Successive wars highly increased TB cases and related deaths (278/100,000 in 1999 versus 321/100,000 in 2002, for example)	[8,9]
Somalia	Highest-ever documented MDR-TB rates in Africa and the Middle East (5.2% and 40.8% of newly diagnosed and previously treated cases, respectively)	[10]
Ukraine	A significant increase in M/XDR-TB cases (14% before the war versus 25% in 2016 according to WHO)	[11]
**Refugees or displaced populations**		
Ethiopian and Chadian refugees in Sudan	TB caused 30 to 50% of total deaths	[12]
Ethiopian refugees in Somalia	26% of adult mortality was caused by TB	[12]
Internally displaced Salvadorians	The estimated incidence of smear-positive pulmonary TB was 125 per 100,000, or 3 times the national reported rate for El Salvador	[13]
Tibetan refugees from India and Nepal in Minnesota, United States of America	LTBI is found among 98% of the Tibetan refugees; high prevalence of active TB cases (8,377/100,000) and MDR-TB cases (19% of active TB cases)	[14]
Tibetan refugees in India	Highest TB incidence in the world (835–1,700 cases/100,000 individuals in the mid-1990s); MDR estimates much higher than the host country (14.5% and 31.4% of MDR-TB cases, respectively, among new and previously treated cases)	[15–17]
Somali, Ethiopian, and Sudanese refugees in Kenya	Resistant TB forms represent 18.3% versus 5.7% in refugee and nonrefugee populations, respectively	[18]
Tibetan refugees in Toronto, Canada	Prevalence of active TB cases much higher than overall Toronto (4,571/100,000 versus 20–25/100,000); MDR-TB cases much higher than the host city (17% vs. 2% respectively)	[19]
Internally displaced Georgians	Prevalence of active TB cases is more than twice than is reported for the entire Georgian population (537/100,000 versus 200/100,000)	[20]
North Korean refugees in the Republic of Korea	MDR rates are higher in refugees than in host population (23% versus 2.4%, respectively)	[21]
US-bound Hmong refugees in Thailand	MDR-TB outbreak in the camp in Thailand; importation of resistant strains to the US by US-bound refugees	[22]
Migrants to Europe in recent years	MDR-TB is more prevalent among migrants than the native population in low-incidence European countries	[23]

LTBI, latent tuberculosis infection; MDR-TB, multidrug-resistant tuberculosis; TB, tuberculosis; WHO, World Health Organization; XDR-TB, extensive drug-resistant tuberculosis.

## Increasing risk of TB development among Syrian citizens and SRs in neighboring countries

The Syrian conflict, now in its eighth year, is the largest humanitarian emergency since World War II. This bloody conflict has had a marked devastating impact on the country’s healthcare infrastructure, sanitation, and medical services. According to Physicians for Human Rights (PHR), there were 478 attacks on 323 separate facilities, and at least 826 medical personnel were killed between March 2011 and the end of June 2017.

Before the onset of the Syrian crisis, the national TB prevalence had decreased from 85/100,000 in 1990 to 23/100,000 in 2011. Since the beginning of the war, because of the emphatic destruction of facilities and the scarcity of microbiological diagnostic tests, the true TB prevalence remains elusive, and reported cases represent only the tip of the iceberg [24]. However, in 2015, WHO recorded 2,992 reported TB cases, with an estimated rate of MDR-TB of 8% of new cases and 18% of previously treated cases. In August 2017, the Syrian Ministry of Public Health declared 3,820 pulmonary TB cases in their official data on health indicators.

Unfortunately, the Syrian war has pushed millions of Syrians to seek refuge in neighboring countries, including Lebanon, Turkey, Jordan, and Iraq. In December 2017, the Office of the UN High Commissioner for Refugees (UNHCR) reported that there were about 5.5 million SRs in these countries. Regrettably, a clear increase in TB prevalence among this population has been documented in neighboring countries, thus contributing to a rise in the number of disease cases across the region [25].

According to the UNHCR, Turkey sheltered the largest number of SRs, reaching over 3,561,707 as of December 2017. Although the TB incidence in this country is progressively decreasing, the Turkish Ministry of Health reported a noticeable increase in the proportion of imported TB cases relative to the total TB cases with the beginning of Syrian unrest: from 1.3% in 2011 to 6.8% in 2015. Screening for TB in 10,689 SRs identified a prevalence of 18.7/100,000 [26]. As of October 2015, 558 new TB cases among SRs were detected and treated in Turkey [26]. Additional data from Hatay province, a neighbor city of Syria and hosting a substantial number of SRs, demonstrated an increase in pulmonary TB cases from 25.1% to 34.6% within a 4-year period (2010–2013) [27].

According to UNHCR, Jordan is hosting about 659,063 SRs (December 2017). In 2010, TB prevalence in this country was 6 cases/100,000 people [25]. From March 2012 through June 2013, 59 TB cases were identified among SRs [28], and it was assumed that almost 22% of TB cases reported in Jordan would be among this population [25]. This increasing number of reported TB cases in Syrians nationwide seriously postponed Jordan’s TB elimination program [29]. Therefore, the Jordan National TB Program (NTP), in collaboration with several international partners, implemented in July 2013 a specific public health strategy among SRs in Jordan to target TB reduction in this vulnerable population.

In Lebanon, according to the country officials, the current total number of SRs is estimated to be more than 1.5 million, representing over a quarter of the national population. Contrary to Turkey and Jordan, there are no formal refugee camps in Lebanon, and refugees are distributed in several hundreds of sites across the nation [25,30]. Referring to WHO and the Lebanese Ministry of Public Health, the trend of TB incidence in Lebanon had been declining until 2011. However, since this date, which coincides with the beginning of the Syrian conflict, accumulating data underline a remarkable increase in TB cases in Lebanon and indicate that this is essentially attributable to the massive displacement of the SR population [25,31,32].

In 2010, before the Syrian crisis, the non-national/national percentage of TB cases in Lebanon was 33%. However, this percentage started to increase, gradually reaching 53% in 2015. This fact, along with the increase of total TB cases in Lebanon after the beginning of the Syrian crisis in 2011, reveal that the huge displacement of SRs constitutes an emerging risk factor of TB development and spreading in Lebanon. In line with this, a more recent report published in 2015 and carried out by WHO, the International Organization for Migration (IOM), and the ministry of public health of Lebanon showed that, among notified TB cases, the proportion of Lebanese nationals with TB declined from 66.7% to 49.4% between 2010 and 2014, while that of SRs increased 10-fold: from 1.6% to 16% in the same period [33]. In 2016, 21.6% of notified TB cases in Lebanon were detected among SRs (Table 2).

**Table 2:** Trend over time of the number of notified TB cases by origin in Lebanon during the 2010–2016 period.

	2010	2011	2012	2013	2014	2015	2016
Total reported TB cases*	515	498	630	689	682	666	679
**National TB cases**	344	298	330	341	337	312	321
**Non-national TB cases**	171	200	300	348	345	354	358
Syrian refugee TB cases**	8***	15	41	106	109	139	147
**% of total cases represented by Syrian notified cases**	1.6%	3%	6.5%	15.4%	16%	21%	21.6%

* Total notified TB cases represent the sum of those national and non-national.

**Syrian refugee TB cases represent the number of Syrian TB cases among the non-national ones.

***This number is detected among resident Syrians in Lebanon before the onset of the Syrian war. Data obtained from [31–34]. TB, tuberculosis.

## Low treatment success rates and drug-resistant TB spreading among SRs

In 2010, we reported that MDR-TB strains are abundantly scattered across Syria [35]. Among the 88 Mtb clinical isolates we tested, 52 (62.5%) were MDR strains. Unfortunately, the Syrian war circumstances force TB patients to interrupt their treatment [30]. Moreover, several among them who leave Syria and take refuge in neighboring countries face personal instability and difficult living conditions that may obstruct treatment possibilities. This decreases the treatment success rate and represents a high risk of drug resistant-TB development. In this context, the Lebanese national TB program data reveal that treatment success remains below the desired rate among non-Lebanese patients, with almost 50% of patients in these groups leaving the country before the completion of their treatment [32]. In Turkey, a lower treatment success rate (63.6%) was noted in SRs compared to Turkish citizens (88.8%) [27]. Concerning MDR-TB, although reported cases are few in Lebanon, national health authorities underline that they may increase among SRs that could not have regular treatment [32]. In 2013, among TB cases notified in SRs in Lebanon, about 3% were MDR-TB [34]. In Jordan, 3 MDR-TB cases (5%) were reported among the total of 59 notified cases from March 2012 through June 2013 [28]. Taken together, these data reflect an increasing potential risk of MDR-TB spreading in countries hosting SRs in the future.

## A health threat and a need for coordinated national and international efforts

The miserable conditions of the ongoing Syrian conflict and related massive displacement of SRs highly promote the risk of TB development, spreading, and resistance among Syrian citizens as well as SRs and their host communities. This represents a big challenge for the national fight against TB in these overburdened countries. Consequently, countries hosting SRs must implement efficient preventive and curative national measures. Preventive actions include firstly the improvement of SR living, sanitary, and health conditions in order to limit TB-associated risk factors. Secondly, health authorities must recommend the BCG vaccine, which is optional in some hosting countries, with national mandatory vaccines to both SR children and their citizen counterparts. Thirdly, successive monitoring epidemiological surveys aiming to determine the prevalence of LTBI among SRs are also required to evaluate and subsequently, when possible, treat latent Mtb infection in this high-risk population. The curative actions need first to identify potential TB patients, and this represents a pivotal difficulty because of the wide refugee distribution. Once identified, probable patients must be accurately and rapidly diagnosed and treated to limit disease progression and further contagion. This requires that health systems insure and support advanced reliable diagnostics (e.g., Xpert MTB/RIF tests) and cover the costs of anti-TB medications. Moreover, to avoid disease spreading and resistance, efficient follow-up systems of TB-identified and treated refugees must be set up in this unstable population. Finally, it is worthwhile to remember that dire conditions faced by SRs in neighboring countries pushed them to leave and illegally migrate toward many other distant countries. This may create a global challenge in the battle against TB and may favor dissemination of the disease and its resistant forms across international borders. Consequently, coordinated efforts to prevent, treat, and limit TB spreading in this highly vulnerable population must be a global health crisis. Such efforts require the engagement of a variety of worldwide partners and are pivotal in promoting the accomplishment of the ambitious targets of WHO’s new post-2015 Global TB Strategy, the End TB Strategy.
